# Potential roles of *Culicoides* spp. (*Culicoides imicola, Culicoides oxystoma*) as biological vectors of bluetongue virus in Yuanyang of Yunnan, P. R. China

**DOI:** 10.3389/fcimb.2023.1283216

**Published:** 2024-01-11

**Authors:** Nan Li, Jinxin Meng, Yuwen He, Wenhua Wang, Jinglin Wang

**Affiliations:** ^1^ Yunnan Tropical and Subtropical Animal Viral Disease Laboratory, Yunnan Animal Science and Veterinary Institute, Kunming, China; ^2^ The Aquaculture Workstation of Yuanyang County Agriculture, Rural Affairs, and Science and Technology Bureau, Yuanyang, China

**Keywords:** *Culicoides*, molecular identification, blood-meal source, Bluetongue virus, African horse sickness virus

## Abstract

**Introduction:**

*Culicoides* plays a crucial role as an insect vector in the field of veterinary medicine. The transmission of significant viruses such as bluetongue virus (BTV) and African horse sickness virus (AHSV) by this insect poses a substantial threat, leading to the development of severe diseases in domestic animals. This study aimed to explore the *Culicoides* species, identify their blood-meal sources, and assess the presence of BTV and AHSV carried by *Culicoides* in Yuanyang County, Yunnan Province. The aim was to gain insights into the potential vectors of these two viruses and elucidate their potential roles in the transmission of pathogens.

**Methods:**

The midges were collected from cattle (*Bos indicus*), pig (*Sus scrofa*), and goat (*Capra hircus*) pens in Yuanyang County, Yunnan Province in June 2020. Initial identification of midges was conducted through morphological characteristics, followed by molecular identification using the cytochrome C oxidase subunit I (COI) gene. The determination of *Culicoides* blood-meal sources was accomplished using specific primers targeting the cytochrome *b* (Cyt *b*) gene from potential hosts. BTV and AHSV RNA were identified in *Culicoides* pools through the application of reverse transcriptase PCR and quantitative real-time PCR. Nucleotide homology and phylogenetic analysis were performed using MegAlign (DNAStar) and Mega 6.0 software.

**Results:**

A total of 6,300 *Culicoides*, consisting of *C. oxystoma, C. arakawai, C. imicola*, and *C. innoxius*, were collected from cattle, pigs, and goat pens. The engorgement rates for these species were 30.2%, 54.6%, 75%, and 66.7%, respectively. In the cattle pen, the prevailing species is *C. oxystoma* (100%). In the pig pen, *C. arakawai * dominates (70%), with *C. oxystoma* following at 30%. In the goat pen, *C. imicola* holds the majority (45.45%), trailed by *C. oxystoma* (25%), *C. innoxius* (20.45%), and *C. arakawai* (9.09%). These *Culicoides* species were identified as feeding on cattle, pigs, goats, chickens (*Gallus gallus*), and humans (*Homo sapiens*). The positivity rates for BTV were 20.00% and 11.54% in blood-fed specimens of *C. imicola* and *C. oxystoma*, respectively. Conversely, the positivity rates for BTV in non-blood-fed specimens were 0.00% and 6.67% for *C. imicola* and *C. oxystoma*, respectively. BTV was not detected in *C. arakawai* and *C. innoxius*. The specimens (YY86) from *C. imicola* that tested positive for BTV had the closest genetic relationship to YTS-4 isolated from Mangshi, Yunnan Province in 1996. All test results for the nucleic acid of AHSV were negative.

**Conclusion:**

The study reveals variations in the species distribution, community composition, blood sucking rate, and blood-feeding sources of *Culicoides* across different habitats. Notably, *C. imicola* and *C. oxystoma* emerge as potential vectors for the transmission of BTV in local animals. Accordingly, this investigation provides crucial insights that can serve as a valuable reference for the prevention and control of BTV in local animals, particularly from the perspective of vector management.

## 1 Introduction

Bluetongue virus (BTV) and African horse sickness virus (AHSV) are globally recognized as two distinct orbivirus that bear the closest association with animal diseases and have a significant impact on animal husbandry and veterinary health. These orbiviruses pose a serious threat to economically valuable livestock species, including cattle (*Bos indicus*), goat (*Capra hircus*), pig (*Sus scrofa*), and horses, often leading to high mortality rates in affected animals. For example, the death rate of bluetongue (BT) in goat and African horse sickness (AHS) in horses can reach as high as 75% and 90%, respectively (Coetzer and Guthrie, 2004; Najarnezhad and Rajae, 2013; Carpenter et al., 2017). Initially confined to Africa and parts of Europe, these two animal diseases have witnessed a surge in prevalence in recent years. This escalation is attributed to factors such as social development, increased transportation networks, and rise in livestock exchanges and trade. Consequently, there has been a significant upswing in the transmission and spread of *Culicoides*, the vector responsible for the BTV and AHSV in recent years. Not only has the intensity of these epidemics increased, but their geographical scope has also expanded continuously, often leading to trans-regional and trans-continental outbreak and a large number of animal fatalities and resulting in significant economic losses (Alkhamis et al., 2020).

*Culicoides* plays an important role in the transmission of BTV and AHSV, and the occurrence and spread of BT are closely linked to the species composition and distribution of potential *Culicoides*. The genus *Culicoides* comprises a global total of 1,399 species (Borkent and Dominiak, 2020), but only a limited number have been implicated with the transmission of BTV and AHSV. Notable species proven to be vectors of BTV include *C. imicola*, *C. obsoletus*, *C. schultzei*, *C. wadai*, *C. fulvus*, *C. brevitarsis*, *C. variipennis*, *C. insignis*, *C. actoni*, *C. oxystoma*, *C. orientalis*, and *C. peregrines* (Sendow et al., 1996; Mellor et al., 2000; Venter et al., 2000; Dadawala et al., 2012). Additionally, species such as *C. oxystoma*, *C. imicola*, and *C. bolitinos* have been identified as potential vectors of AHSV(Meiswinkel and Paweska, 2003; Moussa et al., 2015). The distribution and ecosystem of these species of *Culicoides* significantly influence the occurrence, spread, and severity of both BT and AHS. A comprehensive understanding of the species composition, distribution, blood-meal host of *Culicoides*, and the arbovirus they carry is the key to effectively prevent and control the epidemic and transmission of BT and AHS.

The initial report of a BT outbreak in goats dates back to 1979 in Yunnan Province, China. Since then, BT has been identified in animals, including cattle, across various regions of China. Up to now, BTV has been detected in infected livestock in 29 provinces, including Guangdong, Guangxi, and Xinjiang. Ten regions have reported the isolation of 16 BTV serotypes (BTV-1, BTV-2, BTV-3, BTV-4, BTV-5, BTV-7, BTV-9, BTV-11, BTV-12, BTV-14, BTV-15, BTV-16, BTV-17, BTV-21, BTV-24, and BTV-29) from infected goat or sentinel animals (Ma et al., 2017; Qin et al., 2018; Duan et al., 2019; Li et al., 2020; Li et al., 2021). The diverse array of BTV serotypes poses a significant threat to the country’s cattle and goat breeding industry. AHSV primarily affects regions in Africa and the Middle East (King et al., 2020). Notably, *C. imicola*, the primary vector of AHSV, is presented in the southern regions of China, heightening the risk of AHS being introduced to China. Despite this potential risk, there is a notable absence of research on the species composition, blood-meal sources, and arbovirus-carrying vectors associated with both BT and AHS. The aim of this study was to identify *Culicoides* collected from different habitats in Yuanyang, Yunnan Province, utilizing morphological and molecular identification methods. Additionally, the investigation seeks to analyze the blood-meal sources and arbovirus-carrying potential of *Culicoides*.

## 2 Materials and methods

### 2.1 Collection of *Culicoides* specimens

In June 2020, *Culicoides* specimens were collected from feedlots at three different sites using light traps (12 V, 300 mA; Wuhan Lucky Star Environmental Protection, Hubei, China) situated in Yuanyang County, Honghe Prefecture, Yunnan Province. The sites chosen included goat, cattle, and pig feedlots. The goat feedlot (23°12′N; 102°55′E) and cattle feedlot (23°13′N; 102°50′E) are situated in Wubang and Nansha Village, Nansha Town, at altitudes of 230 m and 242 m, respectively. In contrast, the pig feedlot (23°6′N; 102°45′E) is located in Quanfuzhuang Village, Xinjie Town, at a higher altitude of 1,700 m above sea level. *Culicoides* specimens were collected overnight, spanning from 7:00 pm to 7:00 am. The collected specimens were promptly frozen in a −20°C freezer for 20 min to sacrifice. Subsequently, they were preliminarily classified and identified on ice according to their morphological characteristics following the keys described by Yu et al. (2005). Fifty *Culicoides* per tube were dispensed into cell-freezing tubes, recorded and labeled, and then stored in liquid nitrogen. Finally, those samples were transported to our laboratory for further analysis.

### 2.2 Molecular identification of *Culicoides*

Genomic DNA was extracted from *Culicoides* pools using the TIANamp Micro DNA kit (TIANGEN, Beijing) in accordance with the manufacturer’s recommendation and subsequently stored at −20°C. A fragment of the COI gene was amplified using the primers C1-J-1718 and C1-N-2191 (Dallas et al., 2003) and TaKaRa Ex Taq^®^ DNA Polymerase (TaKaRa, Beijing). PCR reactions were conducted in a total volume of 25 µL, comprising 2 µL of DNA template, 2.5 µL of 10×Ex Taq buffer, 2.5 µL of 2.5 mmol/L dNTP mixture, 0.3 µL of Ex Taq, 0.5 µL of 20 µM of each primer, and 16.7 µL of ddH_2_O. The COI amplification process included an initial denaturation of 94°C for 5 min, followed by 35 cycles of 94°C for 30 s, 54°C for 30 s, 72°C for 40 s, and a final elongation step of 72°C for 10 min. The amplified products were subjected to detection via 1% agarose gel electrophoresis, purification, and Sanger sequencing by Tsingke Biotechnology Co., Ltd. (Beijing, China).

### 2.3 Identification of blood-meal sources

DNA was extracted from engorged *Culicoides* and used as a template, and the fragments of the Cyt *b* region of the mitochondrion were amplified. This amplification was achieved through the utilization of universal vertebrate-specific primer Cytb-F and Cytb-R (Townzen et al., 2008), mammalian-specific primer Mammal-F and Mammal-R (Ngo and Kramer, 2003), and species-specific primer Cow121-F and UNREV1025 (Kent and Norris, 2005). Each 25 µL of PCR reaction comprised 2 µL of DNA template, 2.5 µL of 10× Ex Taq buffer, 2.5 µL of 2.5 mmol/L dNTP mixture, 0.3 µL of Ex Taq, 0.5 µL of 20 µM of each primer, and 16.7 µL of ddH_2_O. The PCR protocol involved an initial heating phase of 94°C for 5 min followed by 35 cycles of denaturation at 94°C for 30 s; annealing at 50°C (Cytb-F and Cytb-R), 55°C (Mammal-F and Mammal-R), and 58°C (Cow121-F and UNREV1025); and elongation at 72°C for 45~60 s. A final elongation step was conducted at 72°C for 10 min. The amplified products were subjected to detection via 1% agarose gel electrophoresis, purification, and Sanger sequencing by Tsingke Biotechnology Co., Ltd. (Beijing, China).

### 2.4 Detection of BTV and AHSV in *Culicoides*

Total RNA was extracted from the grinding supernatant of *Culicoides* pools using RNAiso Plus (TaKaRa), following the manufacturer’s recommendations, and stored at −80°C. To detect BTV and AHSV in *Culicoides* pools, specific primer pairs and TaqMan probes for reverse transcription quantitative PCR (RT-qPCR) were employed (Hofmann et al., 2008; Guthrie et al., 2013). The reaction mix comprised of 2 µL of RNA template, 10 µL of 2× One Step RT-PCR buffer III;, 0.4 µL of TaKaRa Ex Taq HS (5 U/μL), 0.4 µL of PrimeScript RT Enzyme Mix II, 0.4 µL of ROX Reference Dye II (50×), 0.4 µL of 10 µM of each primer and probe, and 5.6 µL of ddH_2_O. The reactions were conducted using One Step PrimeScript™ RT-PCR Kit (TaKaRa) on a Fast 7500 Real-Time PCR machine (Applied Biosystems, Carlsbad, CA, USA) under the following conditions: 42°C for 5 min, 95°C for 10 s, and 35 cycles of 95°C for 3 s and 60°C for 30 s.

### 2.5 Sequence amplification of BTV Seg-3

For RNA samples positive for BTV nucleic acid, reverse transcriptase M-MLV (RNase H−) (TaKaRa) was employed to synthesize the first-strand cDNA in accordance with the manufacturer’s recommendations. Subsequent PCR reactions were conducted using the primer pair BTV L3-1 and BTV L3-2 (Ohashi et al., 2004). Each 25 µL of PCR reaction included 2 µL of cDNA template, 2.5 µL 10× of Ex Taq buffer, 2.5 µL of 2.5 mmol/L dNTP mixture, 0.3 µL of Ex Taq, 0.5 µL of 20 µM of each primer, and 16.7 µL of ddH_2_O. The PCR protocol involved an initial heating phase of 94°C for 5 min, followed by 35 cycles of denaturation at 94°C for 30 s, annealing at 54°C for 30 s, and elongation at 72°C for 40 s. A final elongation step was performed at 72°C for 10 min. The amplified products were subjected to detection via 1% agarose gel electrophoresis, purification, and subsequent Sanger sequencing by Tsingke Biotechnology Co., Ltd. (Beijing, China). All nucleotide positions were confirmed by independent sequencing reactions in both directions.

### 2.6 Sequence analysis

The sequences of the *Culicoides* COI gene and BTV Seg-3 were analyzed using the MegAlign program within the DNAStar software package. The phylogenetic trees for the COI gene and Seg-3 were conducted using the neighbor-joining (NJ) method through MEGA 6.0 software, with a bootstrap value of 1,000 to ensure robustness. Additionally, the nucleotide sequences of the Cyt *b* gene were compared using Nucleotide BLAST in GenBank to analyze the blood-meal sources of *Culicoides*.

### 2.7 Statistical analysis

The minimum infection rate (MIR) value was expressed as a percentage of the number of positive pools per number of individual *Culicoides* tested (Larska et al., 2013). The differences in the composition of *Culicoides* from different environments, the engorgement rate, and the positive rate of BTV detection in different *Culicoides* species were analyzed by chi-square analyses carried out with the SPSS Statistics 26.0 software package. *p ≤*0.05 was considered statistically significant.

## 3 Results

### 3.1 Trapping and identification of *Culicoides* species

After collecting the specimens of midges, the collected midges were frozen to death in a refrigerator at −20°C, and then the miscellaneous insects were picked out. Under a stereomicroscope, the morphology of the midges was preliminarily identified according to the morphology of the wing spot, size, and color of the midges. Fifty *Culicoides* of the same species collected in the same site were placed in a tube as a pool. They were recorded, marked, stored in liquid nitrogen, and finally transported to our laboratory.

**Figure 1:**
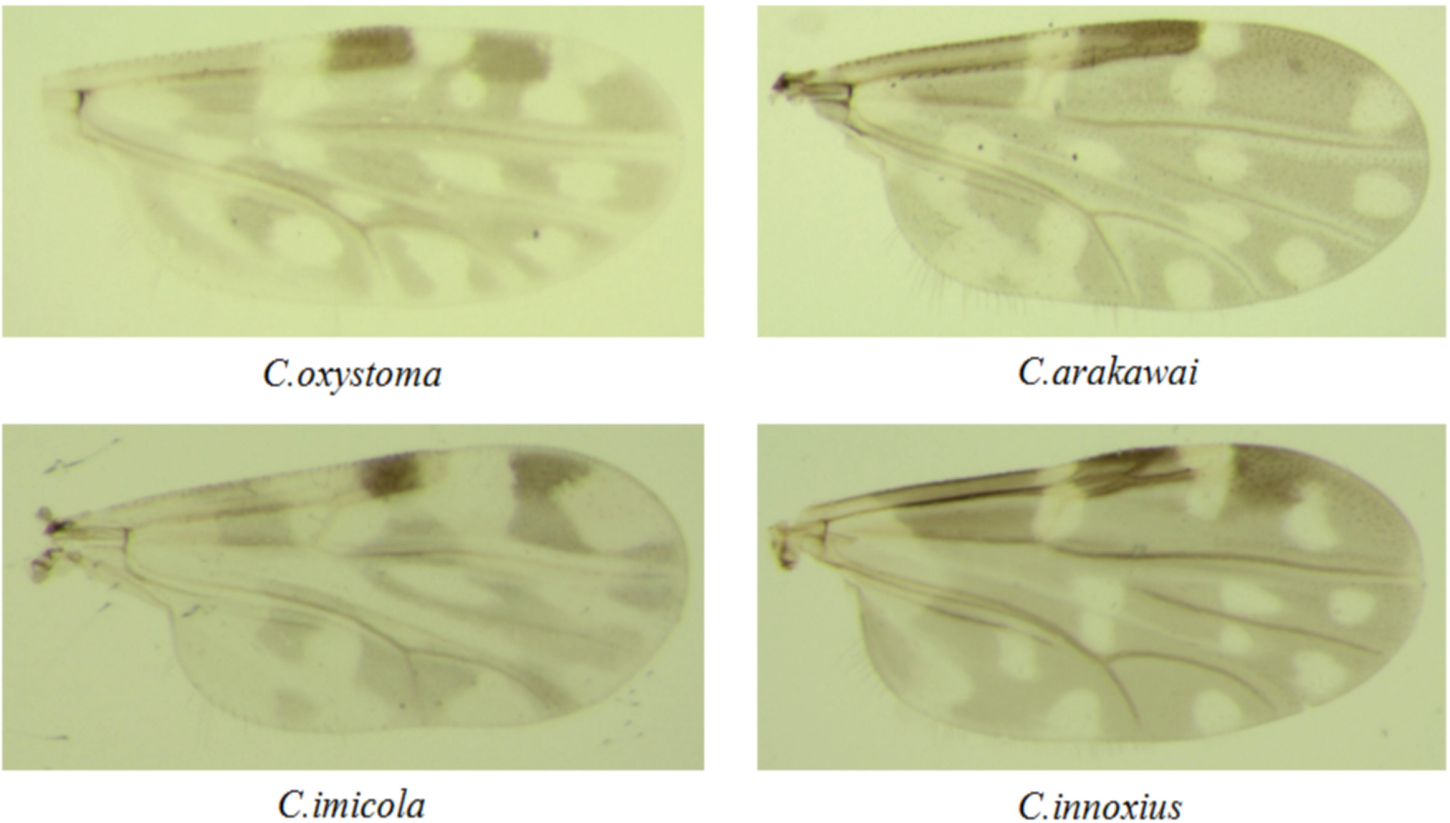
Photographs of the wings of the *Culicoides* species collected in Yuanyang.

**Figure 2:**
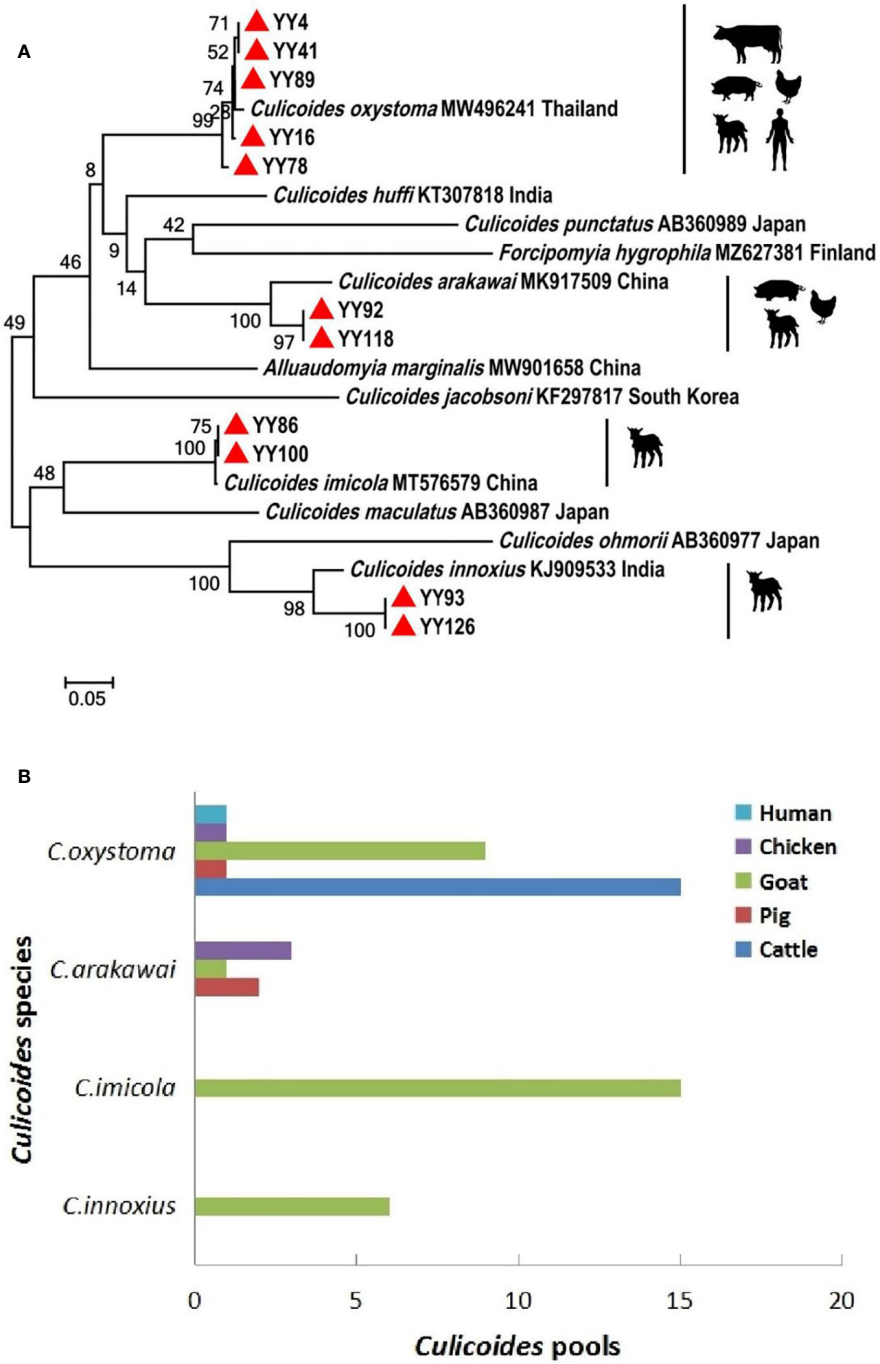
Phylogenetic tree of COI sequences and molecular identification of blood-meal hosts for *Culicoides* species. **(A)** Phylogenetic tree of COI sequences of *Culicoides* and potential hosts of different *Culicoides* species. The specimens created in this study are labeled by solid red triangles. **(B)** Blood-meal hosts were identified from different *Culicoides* species.

A comprehensive collection of approximately 6,300 specimens from the *Culicoides* genus was obtained through light traps set in goat, cattle, and pig feedlots in Yuanyang County, Honghe Prefecture, Yunnan Province. Utilizing both morphological and molecular identification methods, four distinct *Culicoides* species were identified: *C. oxystoma* (68.26%), *C. arakawai* (8.73%), *C. imicola* (15.87%), and *C. innoxius* (7.14%) (**Figures 1**, **2**). The sequences of these *Culicoides* species have been deposited in GenBank with accession numbers (OR472938–OR472948). There were significant differences in the species composition of *Culicoides* collected in cattle, sheep, and pig habitats (*χ*² *=* 6,408.703, *p* < 0.01). *Culicoides oxystoma* emerged as the predominant species in the cattle habitat (100%). In the pig habitat, the dominant species was *C. arakawai* (70%). Within the goat habitat, *C. imicola* (45.45%) was the most abundant species (**Table 1**).

**Table 1:** The species composition of *Culicoides* species in different sites in Yuanyang.

Habitats	*C. oxystoma*	*C. arakawai*	*C. imicola*	*C. innoxius*	Total
	No. (%)	No. (%)	No. (%)	No. (%)	No. (%)
Cattle	3,600 (100)	0 (0)	0 (0)	0 (0)	3,600 (100)
Pig	150 (30)	350 (70)	0 (0)	0 (0)	500 (100)
Goat	550 (25)	200 (9.09)	1,000 (45.45)	450 (20.45)	2,200 (100)
Total	4,300 (68.26)	550 (8.73)	1,000 (15.87)	450 (7.14)	6,300 (100)

### 3.2 Blood-meal source analysis

In total, 6,300 specimens representing *Culicoides* species were collected, with 2,650 of them classified as blood-engorged (42.06%). The collection encompassed various feedlots, including cattle (20.83% blood-fed rate, *n* = 750), pig (40% blood-fed rate, *n* = 200), and goat (77.27% blood-fed rate, *n* = 1,700). There were significant differences in the engorgement rate of *Culicoides* collected in these three environments (*χ*² = 712.825, *p* < 0.001). The engorgement rates of *C. oxystoma*, *C. arakawai*, *C. imicola*, and *C. innoxius* were 30.23%, 54.55%, 75%, and 66.67%, respectively. The engorgement rates of different *Culicoides* species were also significantly different (Fisher’s precision probability test *p* < 0.001).

An effort was made to identify vertebrate hosts for 53 pools of the blood-fed specimens (*n* = 2,650). Initially, all samples were screened with a universal vertebrate primer pair. Subsequently, screening transitioned to a mammalian-specific primer pair and a species-specific primer pair (*Bos taurus* L.) to enhance the identification of specimens. As a result, the total blood-meal host results were obtained through the utilization of several primer pairs. All DNA sequences from blood-engorged specimens showed ≥94% identity matches to vertebrate hosts in GenBank. The origins of the blood-meal host of four *Culicoides* species were identified at the species level. *Culicoides oxystoma* fed on five different vertebrate species: cattle, pig, goat, chicken (*Gallus gallus*), and human (*Homo sapiens*). *Culicoides arakawai* fed on three different vertebrate species: pig, goat, and chicken. In contrast, *C. imicola* and *C. innoxius* exclusively fed on goat (**Table 2** and **Figure 2**).

**Table 2:** Results of blood-meal source analyses in the different *Culicoides* species, according to source (cattle, pig, goat).

Location of traps in relation to livestock	No. of engorged pools (engorged *Culicoides*)	Cyt *b*-positive *Culicoides* pools	*Culicoides* Species	Blood-meal host identified from Cyt *b*-positive *Culicoides* pools (*n*)	Sequence similarity
Trap 1 (near cattle)	15 (750)	100%	*C. oxystoma*	Cattle (15)	99%–100%
Trap 2 (near pig)	4 (200)	100%	*C. oxystoma*	Pig/chicken (1)	100/99.78%
			*C. arakawai*	Pig (2)	99.84%–99.86%
				Chicken (1)	99.11%
Trap 2 (near goat)	34 (1,700)	100%	*C. oxystoma*	Goat (9)	98.41%–99.78%
				Human (1)	99.36%
			*C. arakawai*	Goat (1)	98.75%
				Chicken (2)	98.74%–99.16%
			*C. imicola*	Goat (15)	94.14%–100%
			*C. innoxius*	Goat (6)	99.12%–99.86%

n, number of Cyt *b* sequences.

### 3.3 BTV and AHSV detection

A total of 126 pools, compromising specimens from four *Culicoides* species collected at three distinct sites, underwent testing with BTV- and AHSV-specific RT-qPCR assay. The results of the BTV and AHSV RT-qPCRs for these specimens are summarized in **Table 3**. Among the 86 pools of *C. oxystoma* tested, 26 blood-fed and 60 non-blood-fed specimens were examined, revealing that 3 (11.54%; MIR 0.23%) blood-fed and 4 (6.67%; MIR 0.13%) non-blood-fed pools were identified as infected with BTV, respectively. In the case of *C. imicola*, 15 blood-fed and 5 non-blood-fed pools from the 20 tested pools showed that only 3 (20.00%; MIR 0.4%) blood-fed pools were positive for BTV infection. For *C. arakawai* and *C. innoxiu*s, 6 blood-fed and 5 non-blood-fed and 6 blood-fed and 3 non-blood-fed specimens from 11 pools and 9 pools, respectively, were found to be negative for BTV infection. Additionally, no specimens from the four *Culicoides* species tested positive for AHSV infection.

**Table 3:** Detection of BTV and AHSV carried by *Culicoides* in Yuanyang County.

*Culicoides*	Habitats	No. of blood-fed pools (blood-fed *Culicoides*)	Positive for BTV					No. of non-blood-fed pools (non-blood-fed *Culicoides*)	Positive for BTV				Positive for AHSV
			No. of BTV-positive pools	Proportion of BTV-positive pools (%)	MIR	*Ct* value	Source of blood meal		No. of BTV-positive pools	Proportion of BTV-positive pools (%)	MIR	*Ct* value	
*C. oxystoma*	Cattle	15 (750)	2	13.33	0.27	34.2–34.5	Cattle	57 (2,850)	4	7.02	0.14	34.3–34.9	0
	Pig	1 (50)	1	100	2	31.7	Pig, chicken	2 (100)	0				0
	Goat	10 (500)	0					1 (50)	0				0
Total		26 (1,300)	3	11.54	0.23	31.7–34.5	Cattle, pig, chicken	60 (3,000)	4	6.67	0.13	34.3–34.9	0
*C. arakawai*	Pig	3 (150)	0					4 (200)	0				0
	Goat	3 (150)	0					1 (50)	0				0
Total		6 (300)	0					5 (250)	0				0
*C. imicola*	Goat	15 (750)	3	20	0.4	25.3–34.0	Goat	5 (250)	0				0
*C. innoxius*	Goat	6 (300)	0					3 (150)	0				0

Ct value ≥35 is defined as nucleic acid negative. MIR: percentage of positive pools per number of individual *Culicoides* tested.

### 3.4 Phylogenetic analysis of BTV

**Figure 3:**
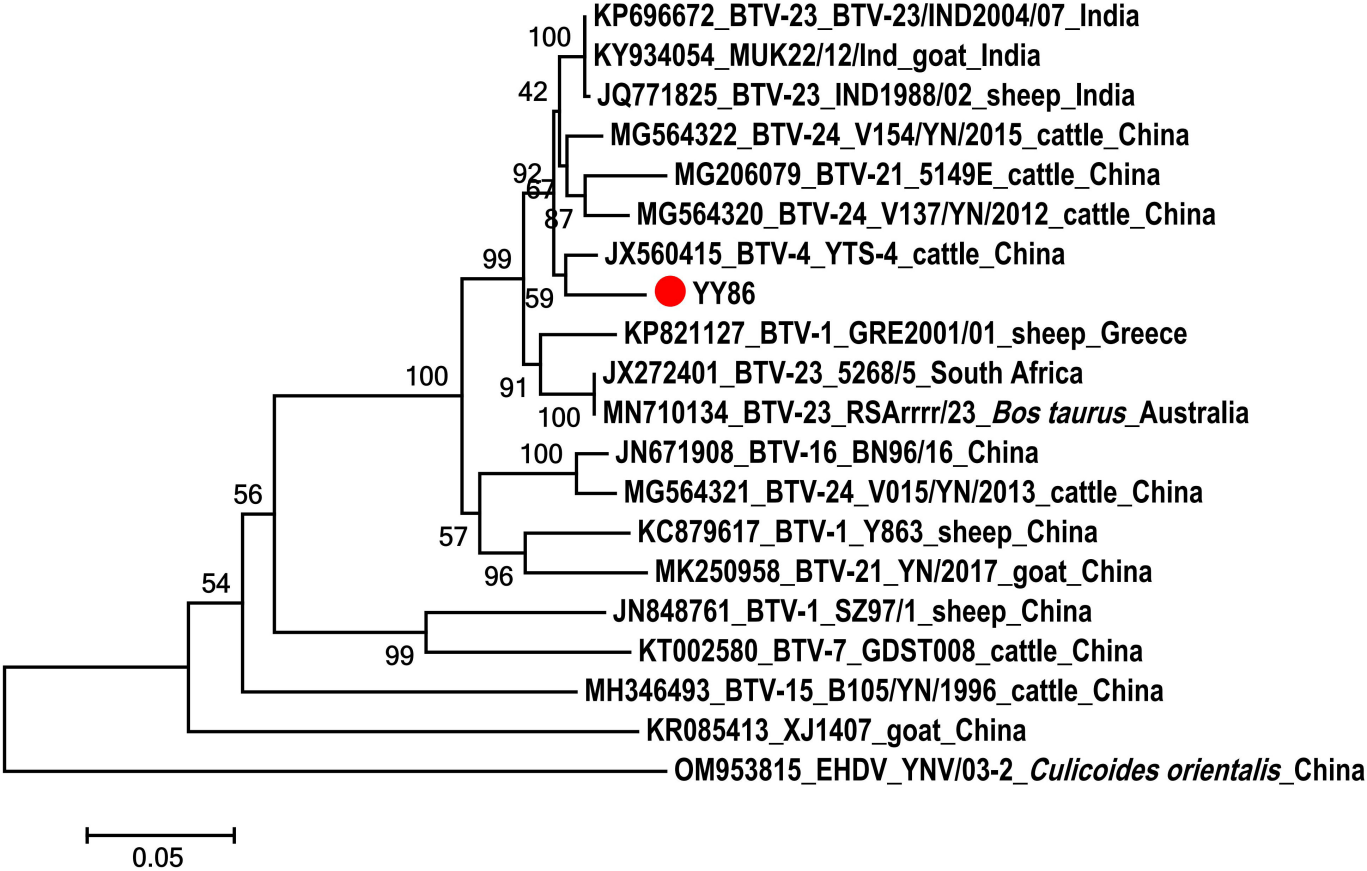
Phylogenetic analysis of the sequences of segment 3 of BTV. The specimens created in this study are labeled by a solid red circle.

A Seg-3 sequence, spanning 688 bp, was derived from the BTV-infected *C. imicola* specimen (YY86) obtained from a goat feedlot. The obtained fragment was utilized to construct phylogenetic trees alongside sequences downloaded from NCBI. As shown in **Figure 3**, YY86 was positioned in the same branch as YTS-4 isolated from Mangshi, Yunnan Province, in 1996, displaying a 96.4% homogeneity (Yang et al., 2012). The data presented here unequivocally affirm that the virus carried by *C. imicola* (YY86) is indeed BTV. The sequence of the BTV Seg-3 has been deposited in GenBank with accession number OR481010.

## 4 Discussion

BT and AHS stand out as the two most severe *Culicoides*-borne livestock diseases affecting ruminants and horses, respectively. BTV and AHSV naturally persist through a series of alternative cycles of replication between *Culicoides* vectors and susceptible hosts. *Culicoides imicola* has been confirmed in previous studies as a principal vector for both BT and AHS, exhibiting a high transmission efficiency for both BTV and AHSV (Braverman et al., 1985; Venter et al., 2000; Paweska et al., 2002; Guichard et al., 2014; de Waal et al., 2016). The widespread distribution of *C. imicola*, spanning the African continent, southern Europe, and extending eastward to South China, poses a significant risk for the transmission and escalation of BT and AHS epidemics (Guichard et al., 2014). In a laboratory setting, *C. imicola* has demonstrated susceptibility to various BTV strains, and it has been established that this vector can also be infected with AHSV (Venter et al., 2004; Venter et al., 2005; Venter et al., 2006a; Venter et al., 2006b; Venter and Paweska, 2007b; Venter et al., 2007a; Venter et al., 2009). South Africa, where *C. imicola* is highly abundant, has reported BTV isolations from field-collected *C. imicola* (Nevill et al., 1992; Meiswinkel et al., 2004). In addition, in Italy, BTV has been detected in *C. imicola* (Goffredo et al., 2015). The presence of BTV in *C. imicola* has also been confirmed in western Thailand and southern Yunnan, indicating the potential significant role of *C. imicola* in BTV transmission (Duan et al., 2021; Fujisawa et al., 2021). Apart from *C. imicola*, *C. oxystoma* is considered a potential vector for arboviruses such as BTV, Akabane virus (AKAV), and epizootic hemorrhagic disease virus (EHDV). This species is geographically widespread in East Asia, Southeast Asia, Australia, and the Middle East (Kitaoka, 1984; Wirth and Hubert, 1989), playing a crucial role in the transmission and distribution of these viruses (Yanase et al., 2005; Dadawala et al., 2012; Bakhoum et al., 2013; Harsha et al., 2020). Dadawala et al. reported the isolation of BTV-1 from non-engorged *C. oxystoma* in India, underscoring the direct involvement of this vector in BTV transmission (Dadawala et al., 2012). Additionally, AKAV has been isolated and detected from *C. oxystoma* in Japan and South Korea (Oem et al., 2013; Kato et al., 2016).

Yunnan Province, situated in the southwest of China, boasts a tropical and subtropical monsoon climate conducive to the survival and reproduction of vector insects and host animals. BTV has been isolated from *Culicoides* or hosts in tropical and subtropical regions of southern Yunnan Province, particularly in epidemic-prone regions (Kirkland et al., 2002; Yang et al., 2016). Yuanyang County, located in the southern part of Yunnan Province, exhibits significant differences in altitude. High-density feedlots in this region house a substantial number of cattle, goats, and other livestock, fostering an environment more conducive to the propagation and transmission of arboviruses. *Culicoides* were collected from three distinct sites with varying elevations in Yuanyang County, encompassing goat (230 m), cattle (242 m), and pig (1,700 m) feedlots, and their composition of *Culicoides* was identified. Four *Culicoides* species (*C. imicola*, *C. oxystoma*, *C. arakawai*, and *C. innoxius*) were identified. Morphological and molecular identification results aligned, confirming *C. imicola* and *C. oxystoma* as potential vectors of BTV. *Culicoides arakawai* has been associated with the transmission of significant livestock pathogens, including BTV, AHSV, AKAV, Chuzan virus (CHUV), and bovine ephemeral fever virus (BEFV) (Yanase et al., 2005; Yang et al., 2018; Kim et al., 2021). The role of *C. innoxius* in disease transmission remains unclear. *Culicoides* activity may be influenced by host distribution. In this study, *C. imicola* was exclusively present in goat habitat, indicating a heightened attraction to goat in the local area. Additionally, topography and climate may impact the abundance and active period of *C. imicola*, favoring hot days in lower-altitude sites. The species’ confinement to low-altitude goat habitats aligns with previous findings (Torina et al., 2004; Conte et al., 2007; Barceló et al., 2021). The highest number of *C. oxystoma* specimens was found across all three sites, with a significant presence in cattle habitats, suggesting an enhanced attraction to cattle. Understanding the local *Culicoides* species and their distribution is pivotal for elucidating their transmission ability and the pathogens they carry. The climate and temperature conditions in Yuanyang County provide an ideal ecosystem for *Culicoides* propagation, coupled with the abundance of susceptible animals, likely facilitating the transmission and spread of BTV.

Locally, livestock serves as the main blood-meal sources of insect vectors, acting as the continuous suppliers of arboviruses. Understanding *Culicoides*’ selection of susceptible hosts is crucial for studying potential vector species (Hadj-Henni et al., 2015; Riddin et al., 2019). Identifying the blood-meal hosts of *Culicoides* is essential to clarify the range of hosts for vectors (Lassen et al., 2011; Leta et al., 2019; Mcgregor et al., 2019). However, compared with mosquitoes and ticks, the blood-meal hosts of *Culicoides* have received relatively less attention (Börstler et al., 2016; Shahhosseini et al., 2018). We employed PCR to amplify the vertebrate host DNA in a blood meal from *Culicoides* collected at various sites, successfully analyzing 53 pools (42.06%) of the blood-fed *Culicoides*. The findings revealed that these four *Culicoides* species mainly feed on mammals, with most blood-meal analysis results aligning with the livestock present at the three sites. *Culicoides* occasionally fed on chickens or humans, suggesting that, in addition to cattle, pigs, and goats, nearby livestock and humans may also contribute to their blood meal. *Culicoides oxystoma* exhibited a diverse range of blood-meal hosts, including cattle, pigs, goats, chickens, and humans. The observation of feeding on susceptible cattle and goats indicated that *C. oxystoma* is likely a potential vector species for BTV and other arboviruses. Furthermore, its broad host preference enhances the potential transmission of pathogens between hosts and domestic animals.

Moreover, significant variations were observed in the species composition and blood-fed rates of *Culicoides* collected at different sites. Notably, the *Culicoides* species collected in goat feedlot exhibited the highest abundance, featuring four *Culicoides* species and a blood-fed rate of 77.27%. In the pig feedlot, two *Culicoides* species were identified, with a blood-fed rate of 40%. Conversely, the cattle feedlot only yielded *C. oxystoma*, with the lowest blood-fed rate at 20.83%. These findings imply that *Culicoides* are more attracted to goats than other domestic animals, such as cattle and pigs. It is noteworthy that despite the relatively small total number of *C. imicola*, it boasted the highest blood-fed rate at 75%. Following closely were *C. innoxius* and *C. arakawai*, with blood-fed rates of 66.67% and 54.55%, respectively. This suggests that *C. imicola* may exhibit stronger vector competence than the other three species. The blood-meal source of the arthropod plays a pivotal role in pathogen transmission and maintenance in natural systems. Identifying the blood-meal sources from engorged *Culicoides* indirectly provides more host information related to *Culicoides*, elucidating their potential host range and activity information. This information is crucial for understanding pathogen transmission and disease risk.

The study was conducted to determine the significant presence of *Culicoides* around livestock, particularly cattle and goats. These *Culicoides* exhibited a preference for feeding on cattle and goats, which are susceptible to BTV, suggesting a potential risk of pathogen transmission and spread among susceptible species. Further BTV tests demonstrated that the positive rates of BTV were 15.00% and 8.14% in *C. imicola* and *C. oxystoma*, respectively. This confirmed the indirect involvement of these two *Culicoides* species in BTV transmission among local domestic animals. Notably, BTV was detected in blood-fed *C. imicola*, and RT-PCR amplification and sequence analysis of YY86 indicated that this BTV had the closest genetic relationship with YTS-4 isolated from Mangshi, Yunnan Province in 1996. This study detected BTV in *C. imicola* and *C. oxystoma*, suggesting that they are potential vectors of BTV in this region. Hence, local livestock should be vaccinated using several licensed commercial vaccines, including modified live (live-attenuated) and inactivated products. However, all test results for AHSV were negative, indicating that, despite the presence of suitable insect vectors, no introduction of AHSV was identified in the local area at the moment.

The presence of BTV RNA detected in *Culicoides* in this study has been previously demonstrated in Yunnan Province (Duan et al., 2021). Here, we identified four species of *Culicoides* and used DNA sequencing to directly link *Culicoides* species with blood-feeding on hosts in Yuanyang. Further analysis of blood-meal hosts and virus detection data was conducted, linking *C. imicola* and *C. oxystoma* with the transmission of BTV among local livestock. These findings offer crucial insights for the prevention and control of BTV in the region. It will be very meaningful for the prediction and prevention of BTV in other endemic areas in China. We recommend future surveillance studies of potential vectors related to BTV in other parts of China.

## Data availability statement

The datasets presented in this study can be found in online repositories. The names of the repository/repositories and accession number(s) can be found below: https://www.ncbi.nlm.nih.gov/genbank/, OR472938–OR472948; https://www.ncbi.nlm.nih.gov/genbank/, OR481010.

## Author contributions

NL: Conceptualization, Data curation, Investigation, Methodology, Writing – original draft, Writing – review & editing. JM: Data curation, Investigation, Software. YH: Investigation. WW: Investigation. JW: Conceptualization, Funding acquisition, Resources, Supervision, Validation, Writing – review & editing.
